# Determining optimal medical image compression: psychometric and image distortion analysis

**DOI:** 10.1186/1471-2342-12-24

**Published:** 2012-07-31

**Authors:** Alexander C Flint

**Affiliations:** 1 Interconnect Medical, LLC, Menlo Park, CA, 94025, USA

**Keywords:** Medical image compression, JPEG, Lossy, Psychometrics, Image analysis

## Abstract

**Background:**

Storage issues and bandwidth over networks have led to a need to optimally compress medical imaging files while leaving clinical image quality uncompromised.

**Methods:**

To determine the range of clinically acceptable medical image compression across multiple modalities (CT, MR, and XR), we performed psychometric analysis of image distortion thresholds using physician readers and also performed subtraction analysis of medical image distortion by varying degrees of compression.

**Results:**

When physician readers were asked to determine the threshold of compression beyond which images were clinically compromised, the mean image distortion threshold was a JPEG Q value of 23.1 ± 7.0. In Receiver-Operator Characteristics (ROC) plot analysis, compressed images could not be reliably distinguished from original images at any compression level between Q = 50 and Q = 95. Below this range, some readers were able to discriminate the compressed and original images, but high sensitivity and specificity for this discrimination was only encountered at the lowest JPEG Q value tested (Q = 5). Analysis of directly measured magnitude of image distortion from subtracted image pairs showed that the relationship between JPEG Q value and degree of image distortion underwent an upward inflection in the region of the two thresholds determined psychometrically (approximately Q = 25 to Q = 50), with 75 % of the image distortion occurring between Q = 50 and Q = 1.

**Conclusion:**

It is possible to apply lossy JPEG compression to medical images without compromise of clinical image quality. Modest degrees of compression, with a JPEG Q value of 50 or higher (corresponding approximately to a compression ratio of 15:1 or less), can be applied to medical images while leaving the images indistinguishable from the original.

## Background

Medical images are increasingly displayed on a range of devices connected by distributed networks, which place bandwidth constraints on image transmission. As medical imaging has transitioned to digital formats such as DICOM and archives grow in size, [1] optimal settings for image compression are needed to facilitate long-term mass storage requirements.

One definition of optimal medical image compression is a degree of compression that decreases file size substantially but produces a degree of image distortion that is not clinically significant. A more conservative definition of optimal image compression would require a degree of image distortion that cannot be perceived by the viewer at all. Other methods that have been used to distinguish degrees of medical image compression include pixel analysis and blinded measurements of diagnostic accuracy [2].

We assessed the crossover point for distortion of grayscale medical images (CT, MR, and XR modalities) by JPEG compression according to two different definitions: (1) the point at which distortion is clinically significant to the viewer and (2) the point at which any distortion can be reliably discriminated by the viewer. We additionally performed analysis of subtracted images to correlate the accumulation of increasing error pixel burden at lower JPEG Q values with the thresholds determined psychometrically.

## Methods

### Test Images

**Figure 1:**
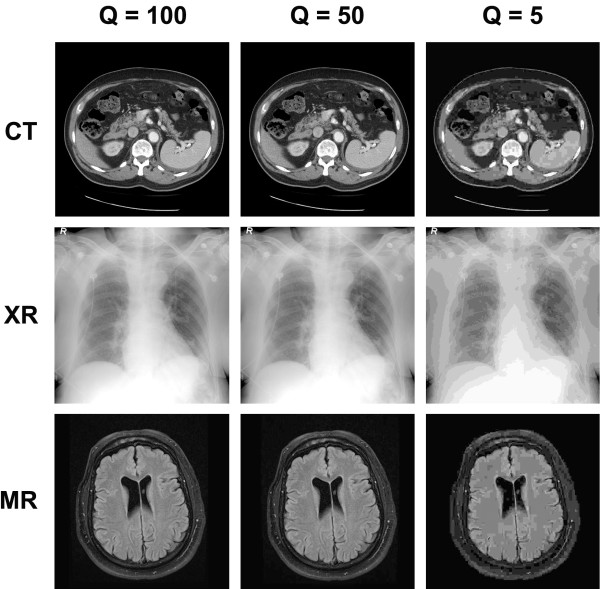
**Example images: JPEG compression.** An example of each of the imaging modalities studied (CT, XR, and MR) is shown at three levels of JPEG Q value (100, 50, and 5).

40 fully anonymized test images without any identifying features in DICOM format were subjected to JPEG compression as described in detail below using ImageJ64 software (version 1.45, http://rsbweb.nih.gov/ij/index.html). Single representative images with or without pathological features were chosen across a range of modalities and body regions, including CT, MR, and XR imaging modalities (Figure 1, Additional file 1). Clinically standard window/level settings for each modality/body region were chosen for presentation. All images were grayscale, at 8-bit depth (0 to 255 gray values), at source pixel dimensions (minimum pixel dimensions 512 x 512, maximum pixel dimensions 2328 x 2320).

### Image Viewing by Clinicians

Because this study aimed to determine thresholds for image distortion by JPEG compression during viewing of images in a range of clinical contexts (e.g. on a personal or clinical office computer, using a web browser, or using a portable electronic device), test images were displayed to subjects using Macintosh and Windows PCs and using both image analysis software and HTML5-compatible web browsers. Because background lux levels can impact radiological image interpretation [3,4], background lux levels were measured using a Mastech MS8229 lux meter and maintained throughout viewing in the range of 25–100 lux.

For the presentation of continuous 100 to 1 JPEG Quality image stacks, images were presented using ImageJ64 software on a Macintosh computer with LCD screen dimensions of 1280 x 800 pixels, with images rendered at full size up to the screen resolution. Image stacks consisted of 100 images created by successively compressing an original single DICOM image into the full range of JPEG compression from JPEG Quality 100 to 1. Viewers were instructed to view the entire range of image compression from JPEG Quality 100 to 1 by scrolling through the image stack continuously using left/right arrows on the computer keyboard or scroll gestures on the computer touchpad. Viewers did not have feedback as to the degree of compression while performing this task; determinations were made solely on the basis of image appearance.

For the presentation of pairwise image comparisons, images were displayed using LCD monitors with screen resolutions of 1280 x 800 to 1280 x 1024 pixels with image presentation by way of an HTML5-compatible web browser (Google Chrome version 15) with images displayed at full size up to the screen resolution. For each pairwise comparison, viewers used the left/right arrows on the computer keyboard to rapidly switch back and forth between the two images being compared.

Clinicians in the study were practicing physicians with board certification in their primary medical specialty (Radiology, Neurology, Neurosurgery, Pulmonary/Critical Care Medicine, and Internal Medicine). A total of 8 clinicians participated in the continuous compression experiment, and a total of 10 clinicians participated in the pairwise image comparison experiment. Clinician subjects were blinded to all aspects of study design and any indicators of image compression other than intrinsic image characteristics.

### Psychometric Measurements

Viewers assessed distortion thresholds in two different experiments: (1) determination of clinically important distortion by assessment of continuous JPEG compression from JPEG Q Value 100 to 1, and (2) determination of the level of compression that can be reliably perceived by the viewer, by assessment of a range of differently compressed image pairs.

For the continuous assessment of JPEG compression, viewers scrolled through stacks of 100 images constructed as described above with a range of JPEG compression from JPEG Q Value 100 to 1. Viewers were asked to determine the approximate point at which the image was felt to be distorted to any clinically meaningful extent, and the Q Value corresponding to this point was recorded. Viewers were allowed as much time as needed to make this determination. Each viewer assessed 40 image stacks.

For the pairwise comparison of images, viewers were shown 7 pairs of images for 8 images randomly chosen from the overall set of 40 images. For each image pair, one image was JPEG Quality = 100 and the other image was JPEG Quality = (5, 20, 35, 50, 65, 80, or 95). Each viewer was shown 7 image pairs presented in randomly chosen order and asked to determine which image of each pair (also presented in randomly chosen order) was the lower quality image. Viewers were instructed to choose an image even if they could not tell the images apart (to guess if required), and also to indicate whether they felt that their choice was a guess or not.

Random choices for image selection and order of image presentation were made with the use of a true random number generator (http://www.random.org).

For ROC plot analysis, sensitivity and specificity were calculated based on correct or incorrect identification of “image 0” or “image 1” from each image pair. Because the presentation of image pairs was chosen by random number generator, the labeling of “image 0” or “image 1” for ROC analysis was randomly chosen and the subject’s response of “image 0” or “image 1” was determined by whether the subject correctly identified the compressed image or not.

### Image Pixel Difference Measurements

To determine the degree of absolute pixel differences between compressed images and a source JPEG Q Value 100 image, we performed subtraction of whole images across the range of JPEG compression from Q Value 99 to 1 using ImageJ64 software. Each successively compressed image was subtracted from the source image, yielding a stack of difference images from (Q Value 100–99) to (Q Value 100–1). Measurements were taken of the total density of difference pixels across each image in the stack, and this operation was then performed on all 40 images viewed by the subject as described above. The mean ± standard deviation total image pixel differences across the 40 images were displayed after normalization to the maximal difference in each image stack.

The conduct of this study was fully compliant with the World Medical Association (WMA) Declaration of Helsinki. Fully anonymized images without any identifying features were shown to physicians who volunteered their own time to participate. No identifying data about the individual physicians was used, stored, or transmitted as part of the study. Based on these specific study characteristics, the study was exempt from IRB review. Exempt status was confirmed by the Kaiser Foundation Research Institute IRB.

## Results

### Psychometric Experiment 1: Clinically important distortion

**Figure 2:**
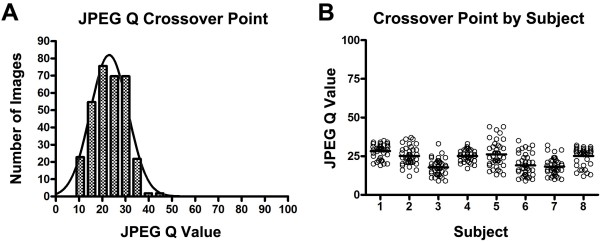
**Analysis of “acceptable image” crossover point.** A. Histogram of n = 320 crossover points from 8 readers, displayed in bins 5 JPEG Q points wide (hatched bars, mean = 23.1 ± 7.0), with a Gaussian curve fit to the data (solid line, mean = 23 ± 8.1). B. Crossover points according to subject. Subjects (1–8) are displayed on the x axis; each data point shows the crossover point for each of the 40 image stacks shown to each subject. Solid horizontal lines show the mean crossover point for each subject.

When physician readers were asked to determine the degree of compression beyond which images were clinically compromised, the mean image distortion threshold was a JPEG Q value of 23.1 ± 7.0 (Figure 2). The distribution of the data about this mean Q value was approximately Gaussian (Figure 2A, mean = 23 ± 8.1). The task in this experiment was a subjective one (determination of the point at which the reader felt the image was unacceptably distorted), so not surprisingly, there was variation in crossover point values from reader to reader (Figure 2B, P < 0.001, Kruskal-Wallis test). Despite this, the highest crossover point for any image or reader was a JPEG Q value of 44.

### Psychometric Experiment 2: ROC plot analysis of discrimination between compressed and original image pairs

**Figure 3:**
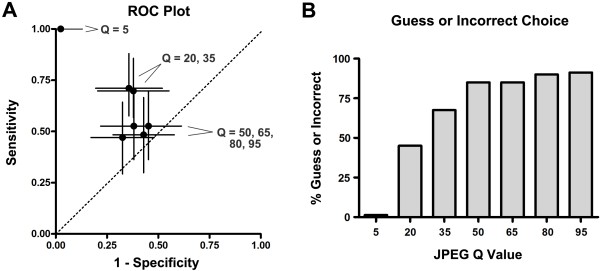
**Pairwise image comparisons: ROC plot and “guess or incorrect” analysis.** A. ROC plot showing sensitivity and (1 - specificity) at each of the levels of compression tested in pairwise (compressed vs. original) image comparisons (Q values of 5, 20, 35, 50, 65, 80, and 95 compared to 100). Point estimates for each Q value (see labels) are solid circles; 95% CI for sensitivity are shown as vertical bars and 95% CI for specificity are shown as horizontal bars. The dotted oblique line represents the “line of unity” where sensitivity = (1 - specificity): points on a ROC plot along this line represent a cut point at which no reliable discrimination has been found. B. Plot of guess or incorrect choice as a function of Q value for the compressed image. The percentage of responses representing an incorrect choice or a known guess are shown at each of the Q values for the compressed image in each randomly presented image pair.

In ROC plot analysis, compressed images could not be reliably distinguished from original images at any compression level between Q = 50 and Q = 95 (Figure 3A). For Q values 50, 65, 80, and 95, the 95 % confidence intervals (CI) of the sensitivity and specificity estimates each crossed the line of unity where (sensitivity = [1 - specificity]), indicating no reliable discrimination between image pairs (Figure 3A). At a Q level of 20 or 35, discrimination between the compressed and original images improved beyond chance (sensitivity and specificity increased and the 95 % CI no longer crossed the line of unity, Figure 3A). However, high sensitivity and specificity for image discrimination was only encountered at the lowest JPEG Q value tested (Q = 5, Figure 3A).

As viewers were additionally asked in this experiment to record whether they felt that their choice was a guess, we also analyzed the relationship between JPEG Q value and the rate at which readers guessed or made the incorrect choice (Figure 3B). Consistent with the ROC plot analysis, the rate of guessing or incorrect choice rose steeply across the Q = 5 to Q = 50 range, then plateaued (Figure 3B).

### Direct analysis of distortion pixels by image subtraction

**Figure 4:**
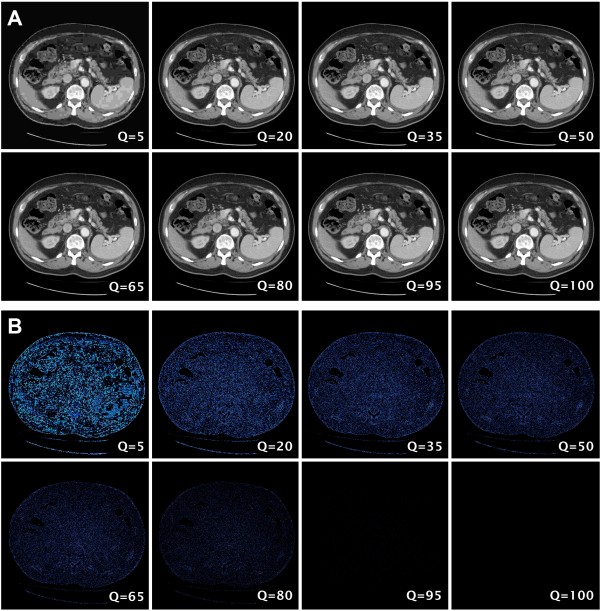
**Visual demonstration of the process of image subtraction to reveal error pixels created by JPEG compression.** A. A range of 8 example JPEG images from an abdominal CT scan, with compression Q values ranging from 5 to 100. B. A range of 8 images created by subtracting each of the images in (A) from the Q = 100 image. If the same window level as the original images in (A) is used, the error pixels are difficult to appreciate, so the images have been pseudocolored with a black to blue lookup table (LUT) and the contrast has been increased to better show the error pixels.

**Figure 5:**
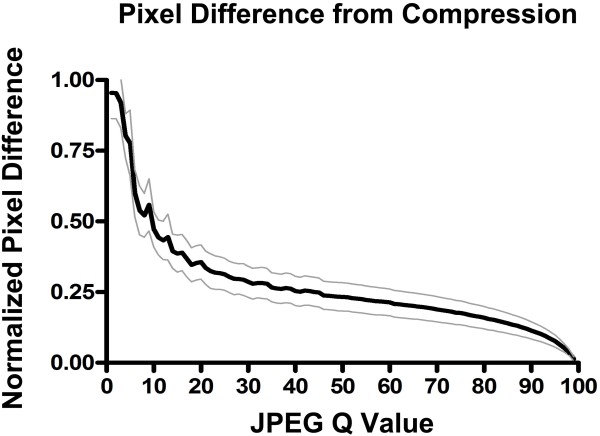
**Normalized average pixel error across the range of JPEG Q values.** Total difference pixel gray values were determined for the range of JPEG Q values from 1 to 99 by subtracting away the original JPEG Q = 100 image and measuring the total value of all difference pixels for each subtracted image. Measurements were averaged after normalization to the maximum difference value for a given difference image stack. The mean normalized pixel difference is shown as a solid black curve flanked by the +/− standard deviation of the mean in solid gray curves.

To determine whether basic features of the JPEG compression algorithm might potentially explain the thresholds encountered in the psychometric experiments above, we performed software analysis of the magnitude of image distortion in subtracted image pairs across the full range of JPEG compression from Q = 99 to Q = 1. A visual demonstration of the effect of image subtraction to reveal error pixels is shown in Figure 4. Direct measurements of subtracted images showed that, as expected, the degree of total pixel error increased across the full range from Q = 99 to Q = 1 (Figure 5). However, this increase in the degree of pixel error had a low slope at Q values above 50, and only at higher levels of compression did the slope show an upward inflection (Figure 5). About 25 % of the error pixel accumulation occurred between Q = 50 and Q = 99, while the remaining 75 % of error pixel accumulation occurred between Q = 1 and Q = 50.

## Discussion

Our data show that lossy JPEG compression can be applied to medical images without clinical image compromise. More subtle lossy JPEG compression (Q values of 50 or higher, roughly a compression ratio of 15:1 or less) can be applied without giving expert viewers the ability to reliably distinguish between the compressed image and the original.

The medical literature on JPEG image compression has typically presented data on compression ratios (e.g. 8:1 or 30:1). However, the software control of compression in the JPEG standard allows for direct manipulation only of Q values, not compression ratio; the compression ratio varies from image to image at a given Q value, depending on the complexity of the source image [5-7]. Since the relationship between Q value and compression ratio for a given image cannot be known *a priori*, it is more reasonable to present data on Q values, assuming software adherence to the standards of the Independent JPEG Group (http://www.ijg.org).

Previous work in this field has focused on relatively subtle degrees of medical image compression. For example, based on a review of the literature on compression of medical images, one group recommended a range of JPEG compression from 5:1 to 8:1. Another review of prior studies recommended this same range of compression.[8] Similarly, consensus-based approaches have yielded estimates of acceptable compression from 5:1 to 15:1 [9]. Another group tested higher degrees of compression following their own literature review[10], but they were unable to perform ROC analysis because the chosen range of compression ratios was too conservative [11]. Of note, in the same study, JPEG compression appeared to perform better than JPEG 2000 compression at the higher levels of compression tested [11]. This observation led us to choose JPEG compression (in contrast to JPEG 2000 compression) for our experiments.

Some work has suggested that higher degrees of compression may be acceptable. For example, one study examined the impact of JPEG 2000 compression on interpretation of mammographic digital images and found that images with compression ratios up to 60:1 were not distinguishable from source images [12].

Our study has limitations. We chose to focus on CT, MR, and XR modalities, all of which are grayscale, and therefore one cannot necessarily extrapolate our results to other imaging modalities, particularly color images. We also chose an approach to determine thresholds of clinically acceptable compression and the ability of readers to discriminate a compressed and original image; therefore, we did not specifically examine the ability of readers to distinguish pathology from normal anatomy, which represents a fundamentally different task.

From the data presented here and data from prior studies,[8,9,11-15] it is reasonable to conclude that a modest degree of JPEG compression is acceptable for many applications, particularly those involving network transmission of images.

## Conclusion

It is possible to apply lossy JPEG compression to medical images (including CT, MR, and XR modalities) without significant compromise of clinical image quality. Regardless of whether one uses a threshold of clinically acceptable quality or a threshold of inability to distinguish the compressed image from the original, use of a JPEG Q value of 50 to 100 (an approximate compression ratio of 15:1 or lower) can be viewed as generally safe. Within the range of JPEG Q values from 50 to 100, trade-offs between quality and file size should be assessed based on the specific application or clinical need.

## Competing interests

The author is Co-Founder and Chief Medical Officer of Interconnect Medical, LLC, a company that designs web-based software for sharing medical imaging.

## Pre-publication history

The pre-publication history for this paper can be accessed here:

http://www.biomedcentral.com/1471-2342/12/24/prepub

## Supplementary Material

Additional file 1 The supplemental video demonstrates the effect of continuously increasing JPEG compression from Q = 100 to Q=1 and then decreasing JPEG compression back up to Q=100 for an abdominal CT scan. The continuous range of Q values shown in the video is similar to what subjects viewed in Psychometric Experiment 1 (see Results), but in the actual experiment, the viewer was able to actively control the process of scrolling through the stack of images. Click here for file
